# Motor interference in interactive contexts

**DOI:** 10.3389/fpsyg.2015.00791

**Published:** 2015-06-11

**Authors:** Eris Chinellato, Umberto Castiello, Luisa Sartori

**Affiliations:** ^1^School of Computing, Faculty of Engineering, University of LeedsLeeds, UK; ^2^Dipartimento di Psicologia Generale, Università di PadovaPadova, Italy; ^3^Cognitive Neuroscience Center, University of PadovaPadova, Italy; ^4^Centro Beniamino Segre, Accademia Nazionale dei LinceiRome, Italy

**Keywords:** action observation, interference effect, movement kinematics, complementary actions

## Abstract

Action observation and execution share overlapping neural substrates, so that simultaneous activation by observation and execution modulates motor performance. Previous literature on simple prehension tasks has revealed that motor influence can be two-sided: facilitation for observed and performed congruent actions and interference for incongruent actions. But little is known of the specific modulations of motor performance in complex forms of interaction. Is it possible that the very same observed movement can lead either to interference or facilitation effects on a temporally overlapping congruent executed action, depending on the context? To answer this question participants were asked to perform a reach-to-grasp movement adopting a precision grip (PG) while: (i) observing a fixation cross, (ii) observing an actor performing a PG with interactive purposes, (iii) observing an actor performing a PG without interactive purposes. In particular, in the interactive condition the actor was shown trying to pour some sugar on a large cup located out of her reach but close to the participant watching the video, thus eliciting in reaction a complementary whole-hand grasp. Notably, fine-grained kinematic analysis for this condition revealed a specific delay in the grasping and reaching components and an increased trajectory deviation despite the observed and executed movement’s congruency. Moreover, early peaks of trajectory deviation seem to indicate that socially relevant stimuli are acknowledged by the motor system very early. These data suggest that interactive contexts can determine a prompt modulation of stimulus–response compatibility effects.

## Introduction

Human beings spend most of their time interacting with others. But despite interest, relevance, and theoretical development on how people represent their own and other person’s actions, there is still a considerable lack of understanding of the precise cognitive mechanisms governing interactive performance. At least part of this remarkable gap is due to the fact that several paradigms have typically relied on single individuals passively observing or imitating other individuals. In contrast, when engaging in interactive contexts, individuals are often required to perform *complementary* parts of a given action, i.e., completing each other’s movement in a balanced manner rather than acting in the same manner (Sebanz et al., 2006; Sartori et al., 2013a). How and when one’s own action execution is influenced by other’s actions execution during social interactions is just beginning to be understood. A large amount of behavioral (e.g., Pfister et al., 2014) as well as neurophysiological studies (e.g., Baess and Prinz, 2015) is providing consistent evidence for the existence of shared representations between action and perception – within and between individuals (Adolphs, 2003; Sebanz et al., 2005). According to the action co-representation account, human agents represent their coactor’s task, and this can *facilitate* action prediction and coordination with others (for a review, see Knoblich et al., 2011).

Others have argued that since one’s own action and the actions of another person are represented in the same way (Hommel, 2009, 2011), actively representing our own and another person’s actions can create a *conflict* between concurrently activated representations (Schubö et al., 2001; Dolk et al., 2014). Concurrent activation during action selection would then produce a discrimination problem, leading participants to emphasize the features that best distinguish selected responses. This implies that more similarity between observed and executed action would put more emphasis on the discriminating features, leading to increased reaction times (RTs) with every extra feature dimension that event-coding processes consider.

The most prominent cognitive paradigms that have been adopted to test these hypotheses in single and joint settings are based on the principle of Stimulus–Response Compatibility (SRC). The term SRC commonly refers to the finding that a compatible mapping of stimulus and response position is associated with shorter RTs as compared to longer RTs due to incompatible mapping (Fitts and Seeger, 1953).

In kinematic terms as well, it has been shown that observing the movements of others can either facilitate or interfere with concurrent movement execution, depending on observed and executed movement congruency (Brass et al., 2000; Castiello et al., 2002; Edwards et al., 2003; Stürmer and Leuthold, 2003; Bertenthal et al., 2006; Liepelt et al., 2010; Hardwick and Edwards, 2012; for reviews, see Blakemore and Frith, 2005; Wilson and Knoblich, 2005). In other words, observing a movement primes the execution of that movement, thereby interfering with the execution of another movement (*motor priming*). Behavioral research on motor priming has shown that responses to human hand movement stimuli (e.g., a hand opening) are faster and more accurate when they involve execution of the same movement (e.g., hand opening) than when they involve execution of an alternative movement (e.g., hand closing; Sturmer et al., 2000). Similarly, if the subjects are instructed to perform a finger tapping in response to a finger tapping (compatible) or lifting (incompatible), the RT to initiate the prepared movement significantly slows down when the stimulus is incompatible (Brass et al., 2001). This effect is thought to be an index of perceptual-motor matching and has been replicated featuring diverse stimulus displays (e.g., grasping, pointing, hand, and arm movements; Kilner et al., 2003) and a variety of stimulus-response arrangements, emphasizing not only the role of perception on concurrent action, but also the influence of movement production over motion perception (Müsseler et al., 2000; Craighero et al., 2002; Hamilton et al., 2004; Schubö et al., 2004; Zwickel et al., 2010; Christensen et al., 2011).

Most importantly for the issue at stake here, Liepelt et al. (2010) found a *reversed compatibility* effect when observing a human extending the right hand for a handshake. When viewing a right-handed shake-hands image, participants responded faster with their own right hand, instead of mirroring the stimulus hand. Notably, we usually shake an extended right hand with our right hand, leading to spatial incompatibility of the relative position of the hand (see also Flach et al., 2010). This reversal of the classic compatibility effect is not surprising in the light of recent finding emphasizing the idea of a complementary action system (Sebanz et al., 2006; Newman-Norlund et al., 2007a,b, 2008; van Schie et al., 2008; Sartori et al., 2013a). It strongly indicates that the overlearned response to extend the right hand when observing a right hand is able to modulate the motor priming effect: when a specific behavior is contingent on a non-matching behavior, an incongruent association is formed (Catmur et al., 2009), so that social response preparation can overwhelm the automatic response (Hamilton, 2013).

The purpose of the present study was to further this line of investigation by exploring how the context specifically modulates actions under ‘complementary’ conditions. The following experiment addressed this issue by adopting ecologically valid stimuli: (i) requiring a specific complementary response (i.e., functionally related to the observed action), (ii) temporally overlapping with the participants’ ongoing action, and (iii) depicting familiar object-oriented hand actions, given that motor familiarity with the observed action is thought to be positively related to the mapping between observed and executed actions (e.g., Calvo-Merino et al., 2005; Cross et al., 2006). We capitalized on an established paradigm for inducing complementary activations in the observers’ muscles (Sartori et al., 2011b, 2012, 2013b,c). In one of these studies (Sartori et al., 2013c), participants watched videos of action sequences showing an actor pouring sugar with a tablespoon, grasped with a precision grip (PG), into a set of cups. At the start of the videos, participants showed a small activation in the little finger muscle, consistent with the actor’s actions. The key manipulation came when the actor stretched the arm toward the last cup, which was placed close to the participant. The socially appropriate response would require to pick up the cup by using a whole hand grip, and offer it to the actor. At this point, the observers’ muscular activations changed, with a large response of the little finger muscle even though the actor in the video maintained the same grip and the participant did not perform any actual response. In the present study, we had participants observing the video of an actor grasping a tablespoon and then stretching toward a cup which was placed close to the participant (interactive action). In another video, the same actor was shown pouring some sugar and then simply coming back to the starting point (non-interactive action). Crucially, the task was to simultaneously observe these perceptual events and perform a congruent prehension (i.e., a PG). Observed and executed action features were thereby maintained compatible across both conditions. By introducing the complementary request by the actor, we expected nonetheless to find an increase of variance in movement trajectory while *planning* an incompatible movement, in line with previous studies demonstrating that trajectory deviations increase when an object is grasped with the intention to interact with a human agent (Becchio et al., 2008a,b; Quesque et al., 2013; Quesque and Coello, 2014). A control condition was also set, in which participants simply observed a fixation cross while performing the task.

Interestingly, previous TMS studies showed *early* changes in observers’ cortico-spinal excitability during observation of hand actions leading to a complementary request (Sartori et al., 2013b,c). More specifically, the changes in cortico-spinal excitability were modulated by early kinematic changes in the observed movement signaling the start of the social request.

Accordingly, in the present experiment we synchronized the ‘go’ signal with the start of the social request (i.e., the time at which the actor finished pouring sugar into the close cup, just before she began stretching her arm toward the out-of-reach cup). We reasoned that if the observer can easily predict the future course of the observed action from the actor’s kinematics, then an *early* motor interference effect should occur on his/her action. In particular, we expected to find a prompt response for the interactive condition in terms of arm trajectory deviation. This would confirm results from a previous kinematic study indicating that socially relevant stimuli are acknowledged by the motor system very early (Sartori et al., 2009). Moreover, since response competition involves inhibition, here we expected to see increased inhibition in the interactive condition, regardless of the fact that the same type of grasp was observed.

## Materials and Methods

### Participants

Fifteen volunteers (nine females and six males, between the ages of 21 and 27) with normal or corrected-to-normal vision participated in the experiment. All the participants were right-handed (Briggs and Nebes, 1975), reported normal or corrected-to-normal visual acuity, and were naive as to the experimental purpose of the study. A right-handed non-professional actor (female, 28 years-old) was also recruited for video clips recording. All participants gave their informed written consent to participate in the study. The experimental procedures were approved by the Institutional Review Board at the University of Padua and were in accordance with the Declaration of Helsinki (Sixth revision, 2008).

### Stimuli

**FIGURE 1:**
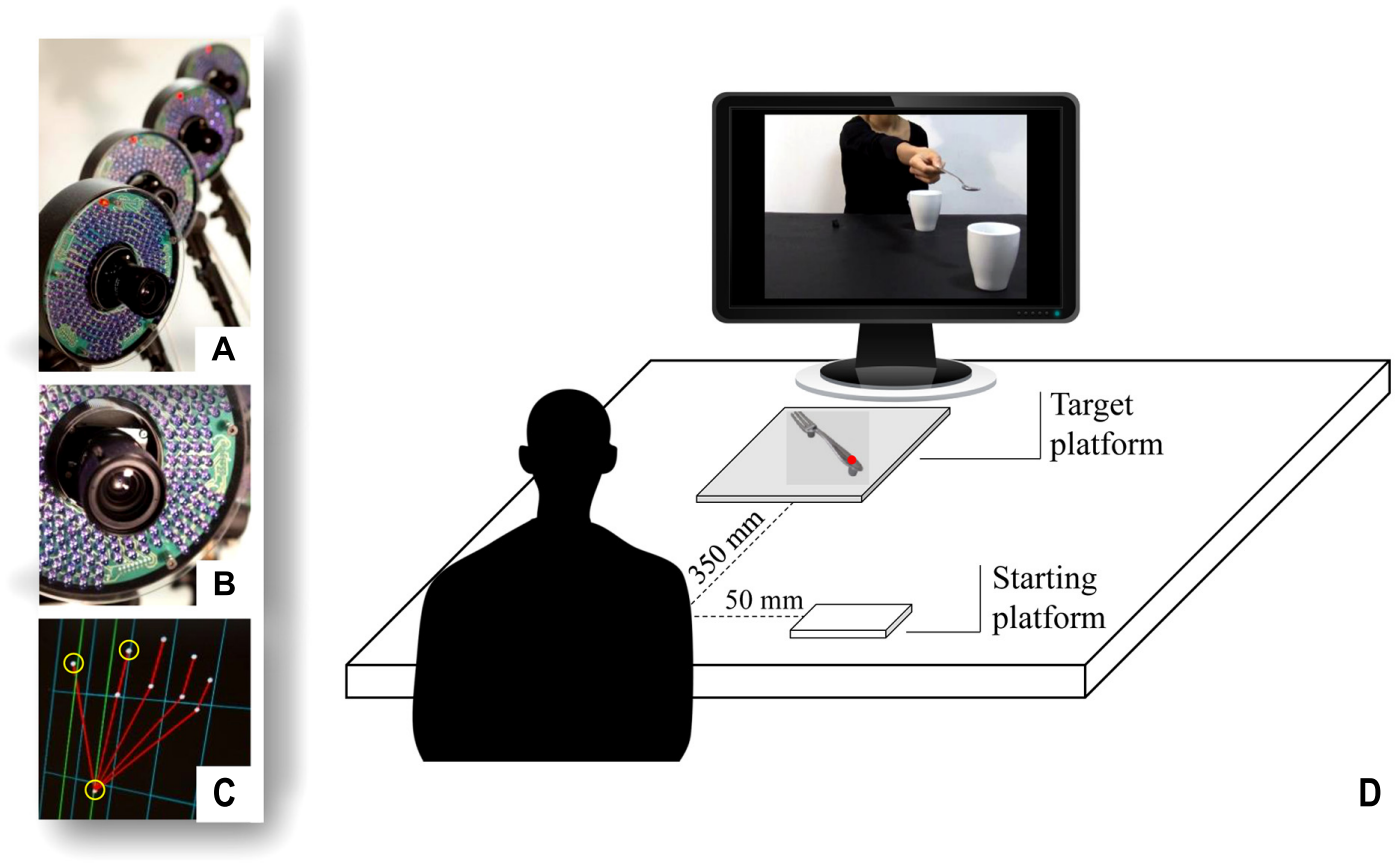
**Set up and procedure**. A 3D-Optoelectronic SMART-D system was used to track the kinematics of the participant’s right upper limb by means of six video cameras **(A,B)**. Infrared reflective markers were taped to the following points: thumb, index finger, and wrist to measure the grasp and reach component of the movement **(C)**. Participants sat in front of a monitor **(D)**. A starting platform was attached 50 mm away from the midsection and the object was placed on a target platform located at a distance of 350 mm. During the interactive condition, the video was showing an actor stretching out her arm trying to pour some sugar on a large cup located close to the participants, thereby inviting them to grasp it.

The stimuli were two digitally recorded video clips showing the actor: (i) pouring sugar with a tablespoon (PG) in a cup located nearby, and then stretching out her arm trying to pour some sugar on a large cup located out of her reach (interactive action; **Figure 1D**), (ii) pouring sugar in the same cup, and then coming back to the starting point (non-interactive action). Crucially, the out-of-reach cup was placed in the video foreground, closer to the participant watching the video, thus eliciting a complementary reaction with a whole-hand grip when the actor was trying to reach for it. All of the videos were taken from a frontal view and were equal in length. At the beginning of each video-clip the hand of the actor was shown in a prone position resting on the table. The model started her reach-to-grasp movement 1 s later and her fingers made contact with the sugar spoon at 4.9 s. The model finished pouring sugar into a close cup 5.8 s after the onset of the video in the interactive condition and 5.9 s in the non-interactive condition. For the participants’ prehension task we adopted a small plastic fork (130 mm length, the same size as the sugar spoon in the videos). An affixed colored dot was signaling which part of the object had to be grasped in order to perform a stable and consistent PG, congruent to the actor’s movement. We choose a fork instead of a spoon to avoid eliciting in the participant the idea of pouring something into the cup – instead of grasping the cup – during the interactive condition. Since gaze is a crucial component of social interactions and could have biased the results, the face of the actor was not visible on the video clips. Eye gaze, in fact, may enhance observers’ abilities in predicting and anticipating others’ actions (e.g., Sartori et al., 2011a).

### Procedure

The experimental set up is depicted in **Figure 1**. Each participant sat on a height-adjustable chair in front of a table (900 mm × 900 mm) with the elbow and wrist resting on the table surface and the right hand in the designated position. The hand was pronated with the palm resting on a starting platform (60 mm × 70 mm; 5 mm thick), which was shaped to allow for a comfortable and repeatable posture of all digits, i.e., slightly flexed at the metacarpal and proximal interphalangeal joints. The starting platform was attached 90 mm away from the edge of the table surface 50 mm away from the midsection. The fork was placed on a target platform (10 cm × 10 cm; 5 mm thick), located at a distance of 350 mm from the starting platform, with the handle pointing slightly rightward (i.e., with an angle of 30° with respect to the midsection) to allow for an accurate prehension. The participants had to execute a reach-to-grasp movement with a PG toward the fork placed on the table and to watch the video clips that were presented on a 19′′ monitor (resolution 1280 × 1024 pixels, refresh frequency 75 Hz, background luminance of 0.5 cd/m2) set at eye level (the eye-screen distance was 60 cm). The experiment included three different conditions:

- Execution-only (control condition): the participants performed their task while observing a fixation cross on the screen.
- Interactive condition: the participants performed their task while observing the video showing an actor performing a PG, and then stretching out her arm trying to pour some sugar on a large cup located close to them, thereby inviting them to grasp it.
- Non-interactive condition: the participants performed their task while observing the video showing an actor performing a PG, and then coming back to the starting point. Notably, in this video the out of reach cup was still visible in the video foreground, therefore controlling for possible affordance effects.

Each condition was presented 15 times in random order. In total, the experiment was composed of 45 trials, each lasting approximately 9 s. Participants were asked to look at the actor’s hand throughout the trials and were instructed to begin their movements as soon as an acoustic ‘go’ signal switched on (‘Go’ instruction). The ‘go’ signal was released 5.8 s after the onset of each video (i.e., the time at which the actor finished pouring sugar into the closely located cup during the interactive condition). Since different attention effects due to different cognitive load in different conditions could have biased our data, we presented the ‘go’ signal when participants were observing the very same gesture (i.e., pouring sugar into the close cup) instead of synchronizing it with the end of the videos. No instruction was given concerning the speed of the movement, and participants were asked to perform their movement at their own pace.

### Kinematics Recording

A 3D-Optoelectronic SMART-D system (Bioengineering Technology and Systems, B| T| S|) was used to track the kinematics of the participant’s right upper limb. Two light-weight infrared reflective markers (0.25 mm in diameter; B| T| S|) were placed on each participant’s hand to measure the grasping component of the action and one marker was placed on the wrist to measure the reaching component of the action (**Figure 1C**, yellow circles). In particular, the three infrared reflective markers were taped to the following points: (i) thumb (ulnar side of the nail); (ii) index finger (radial side of the nail); and (iii) wrist (dorsodistal aspect of the radial styloid process). Six video cameras (sampling rate 140 Hz) detecting the markers were placed in a semicircle at a distance of 1–1.2 m from the table (see **Figure 1**). The camera position, roll angle, zoom, focus, threshold, and brightness were calibrated and adjusted to optimize marker tracking before the trials were begun. Static and dynamic calibration was then carried out. For the static calibration, a three-axes frame of five markers at known distances from each other was placed in the middle of the table. For the dynamic calibration, a three-marker wand was moved throughout the workspace of interest for 60 s. The spatial resolution of the recording system was 0.3 mm over the field of view. The SD of the reconstruction error was below 0.2 mm for the x, y, and z axes.

### Data Processing

Following data collection, each trial was individually checked for correct marker identification and the SMART-D Tracker software package (B| T| S|) was used to provide a 3-D reconstruction of the marker positions as a function of time. The data were then filtered using a finite impulse response linear filter (transition band = 1 Hz, sharpening variable = 2, cut-off frequency = 10 Hz; D’Amico and Ferrigno, 1990, 1992). The measurements were made along the three Cartesian axes [i.e. X (left–right), Y (up–down), and Z (anterior–posterior) axes] of the participants in an upright sitting position. Movement onset was defined as the time at which the tangential velocity of the wrist marker crossed a threshold (5 mm/s) and remained above it for longer than 500 ms. End of movement was defined as the time at which the hand made contact with the object and quantified as the time at which the hand opening velocity crossed a threshold (5 mm/s) after reaching its minimum value and remained above it for longer than 500 ms. The following kinematic parameters were extracted for each individual movement using a custom protocol run in Matlab, 2014b, (The 4 MathWorks, Natick, MA, USA): the time interval between movement onset and end of grasping (Movement Time), the time at which the tangential velocity of the wrist was maximum from movement onset (Time to Peak Wrist Velocity), the time at which the distance between the 3D coordinates of the thumb and index finger was maximum between movement onset and hand contact time (Time to Maximum Grip Aperture), the time at which the tangential velocity of the 3D coordinates of the thumb and index finger was maximum from movement onset (Time to Maximum Grip Velocity), and the maximum distance reached by the 3D coordinates of the thumb and index finger (Maximum Grip Aperture). Grip aperture was calculated at 25, 50, and 75% of the movement to assess during which part of the movement possible interference may occur. In addition, wrist trajectories were computed for each condition, by normalizing each trial according to movement time, so that they were reduced to the same number of time-steps (420). We then considered a spatial trajectory measure that has been proved to be sensitive to variations in social context: the direction, amplitude, and time course of the distance of the trajectory path from a straight line linking the starting position and the object location (Trajectory Deviation; Sartori et al., 2009; Innocenti et al., 2012). For this measure, we gave a positive sign to right deviations and a negative sign to left deviations and we calculated values at 25, 50, 75, and 100% of the movement in both Cartesian distance and signed values. Moreover, temporal delay between the ‘go’ signal and movement onset was computed as RT.

### Data Analysis

The mean value for each parameter of interest were determined for each participant and then entered into separate repeated-measures ANOVAs with Condition (execution-only, interactive, non-interactive) as within-subjects factor. Preliminary analyses were conducted to check for normality, sphericity (Mauchly test), univariate and multivariate outliers, with no serious violations noted. Main effects were used to explore the means of interest (*post hoc t*-test), and Bonferroni’s corrections (alpha level of *p* < 0.05) were applied.

## Results

All the results are displayed in **Table 1**. For the sake of clarity, only parameters differing with respect to interactive vs. non-interactive conditions will be reported. Notably, the fragment of video clip displayed before the go signal (i.e., pouring sugar with a PG) was the same in both these conditions.

**Table 1:** Statistically significant key kinematic parameters and reaction times (RTs) across conditions.

	Execution-only	Interactive	Non-interactive
Movement time (ms)	1287.11 (±61.01)	1339.21 (±64.78)	1336.89 (±62.88)
Time to peak wrist velocity (ms)	535.17 (±29.78)	569.02 (±31.70)	569.24 (±30.28)
Time to peak grip aperture (ms)	681.67 (±46.38)	734.67 (±52.88)	713.18 (±46.12)
Time to peak grip aperture (%)	62.70 (±2.10)	64.70 (±2.00)	64.20 (±1.60)
Time to peak grip velocity (ms)	485.71 (±40.36)	536.86 (±44.44)	510.25 (±36.89)
Trajectory deviation (mm)	5.17 (±1.00)	5.68 (±1.45)	5.08 (±0.91)
Reaction Times (ms)	5437.09 (±305.88)	6425.68 (±184.49)	6093.11 (±173.76)

*Mean and standard errors per condition*.

### Reaction Time

A repeated-measures ANOVA revealed a significant effect of condition [*F*_(2,28)_ = 5.80, *p* = 0.008, $\eta_{p}^{2}$ = 0.29]. *Post hoc* analyses showed that the RT was shorter in the execution-only condition compared to the interactive (*p* = 0.02, $\eta_{p}^{2}$ = 0.31) and non-interactive conditions (*p* = 0.04, $\eta_{p}^{2}$ = 0.27). Moreover, it was more delayed in the interactive condition compared to the non-interactive condition (*p* = 0.05, $\eta_{p}^{2}$ = 0.23).

### Movement Time

The ANOVA performed on movement time revealed a significant effect of condition [*F*_(2,28)_ = 5.72, *p* = 0.008, $\eta_{p}^{2}$ = 0.29]. Observing an interactive action did influence movement performance with respect to the execution-only condition, leading to an increase in the execution time (*p* = 0.01, $\eta_{p}^{2}$ = 0.43). This effect was significant also for the non-interactive condition (*p* = 0.03, $\eta_{p}^{2}$ = 0.31).

### Time to Maximum Grip Velocity

The ANOVA performed on the time of maximum grip velocity revealed a significant main effect of condition [F_(2,28)_ = 10.01, *p* = 0.001, $\eta_{p}^{2}$ = 0.42]. *Post hoc* analyses showed that peak grip velocity was reached earlier in the execution-only condition compared to the interactive (*p* = 0.001, $\eta_{p}^{2}$ = 0.54) and to the non-interactive (*p* = 0.02, $\eta_{p}^{2}$ = 0.34) conditions. Moreover, peak grip velocity was reached later in the interactive than in the non-interactive conditions (*p* = 0.04, $\eta_{p}^{2}$ = 0.26). In normalized terms, a 3% delay of peak velocity for the interactive with respect to the execution-only condition was found, whereas a 2% delay of peak velocity for the non-interactive condition was revealed.

### Trajectory Deviation

**FIGURE 2:**
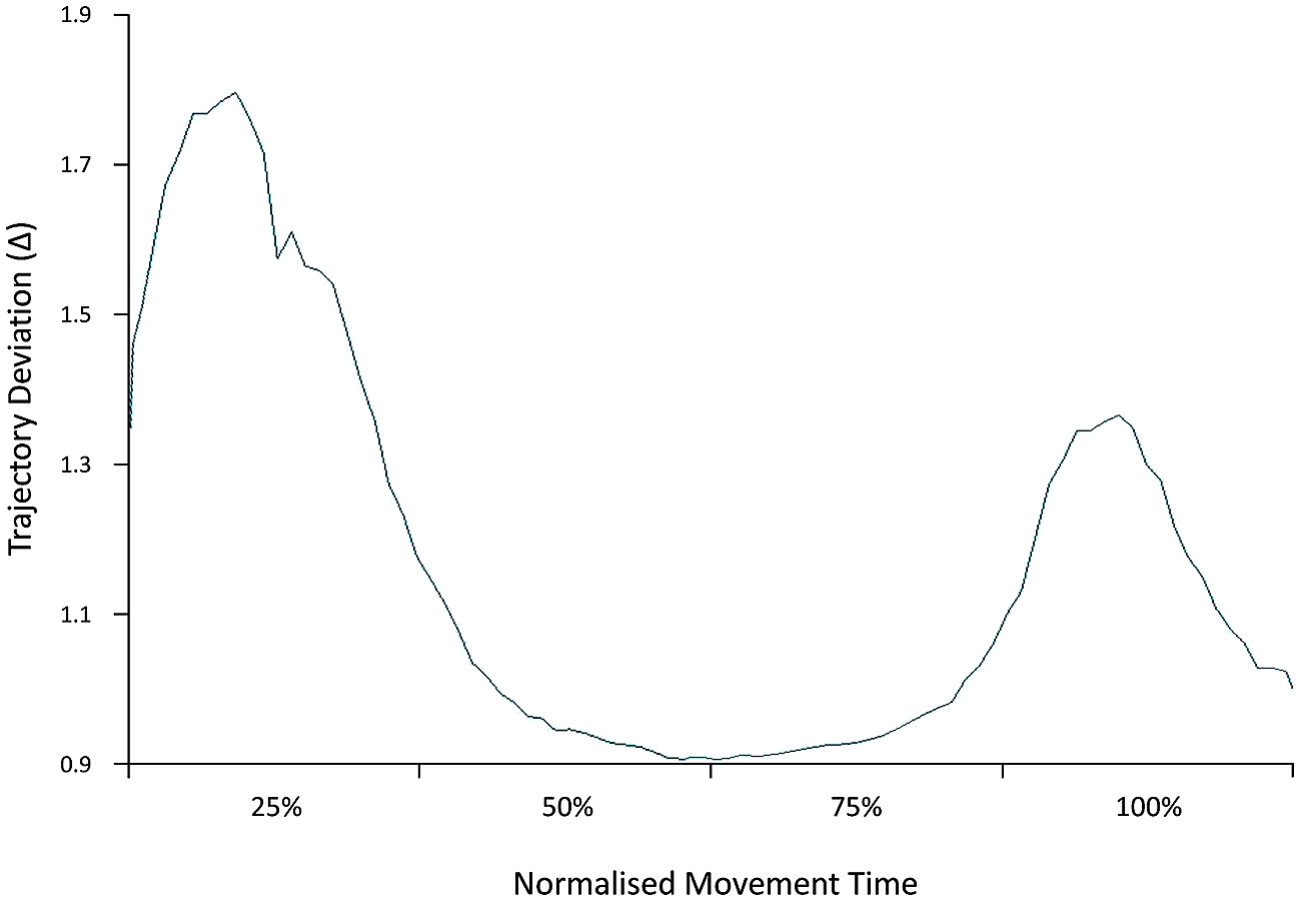
**Early trajectory deviation**. Distance of the trajectory path for the interactive compared to the non-interactive condition is represented at 25, 50, 75, and 100% of the movement. A significant increase in trajectory deviation for the interactive condition was detected within the first 25% of the movement.

The ANOVA performed on the distance of the trajectory path from an ideal straight line linking the starting position and the object location indicates that it was specifically modulated as a function of the condition [*F*_(2,28)_ = 5.32, *p* = 0.01, $\eta_{p}^{2}$ = 0.28]. A significant increase in trajectory deviation was detected for the interactive compared to the non-interactive (*p* = 0.02; $\eta_{p}^{2}$ = 0.36) and to the execution-only condition (*p* = 0.001; $\eta_{p}^{2}$ = 0.33). Notably, when considering the direction and time course of this effect, a statistically significant leftward deviation was detected within the first 25% of the movement for the interactive compared to the non-interactive condition (-1.96 vs. -1.72, respectively; *p* = 0.04, $\eta_{p}^{2}$ = 0.26; **Figure 2**). No effect was found for trajectory deviation at 50, 75, and 100% of the movement (*p*_s_ > 0.05).

### Maximum Grip Aperture

The ANOVA performed on the maximum aperture did not revealed any significant effect of condition [*F*_(2,28)_ = 2.19, *p* > 0.05, $\eta_{p}^{2}$ = 0.14]. However, when considering the time course of grip aperture, a significant decrease was detected for the interactive compared to the execution-only condition at 50% of movement time (41.72 vs. 43.58, respectively; *p* = 0.01, $\eta_{p}^{2}$ = 0.36). No effect was found for grip aperture at 25 and 75% of the movement (*p*_s_ > 0.05).

## Discussion

Many daily activities involve performing an action while simultaneously encoding other perceptual events. This is particularly interesting when others’ actions elicit a complementary response which differ from our ongoing action. The aim of this study was to determine what critical process underlies such mismatching conditions and how they affect the precision and performance of executed movements. Participants were asked to perform a PG with their right hand while concurrently observing a similar action, but requiring (or not) a complementary incongruent response. Our main finding is that although observed and executed action features were maintained compatible across conditions, an increase in RTs, Movement Time, Time to Maximum Grip Velocity, and Trajectory Deviation occurred for the interactive compared to the non-interactive condition, in line with previous studies demonstrating a general delay in the grasping and reaching components and an increased trajectory deviation when an object is grasped with the intention to interact with a human agent (Becchio et al., 2008a,b; Sartori et al., 2009; Quesque et al., 2013; Quesque and Coello, 2014). The very fact that we found a prompt response for the interactive condition (deviation peak at 25% of the movement) indicates that the socially relevant action was acknowledged very early by the motor system (Sartori et al., 2009).

### Reversing Classic Interference Effects

The common coding theory states that perception of an action leads to simulative production of that action on the part of the observer (Brass et al., 2001). But if the central motor system is perfectly tuned during the execution and concurrent observation of a congruent action, what happens when we are required to make a qualitatively different (incongruent) gesture? In this case, the motor program (or representation) associated with the incongruent movement interferes with both the outgoing motor output and the observed movement. And motor interference arises as a general delay in the grasping and reaching components and as an increase of variance in movement trajectory. This result confirms and extends previous findings reporting interference effects when simply observing incongruent moving stimuli presented either face-to-face or in video (Kilner et al., 2003). Moreover, it generalizes the results of Sartori et al. (2009) and Liepelt et al. (2010) showing that planning a complementary, functionally related action has the power to elicit associated responses and reverse classic interference effects. Depending on its posture and context, an extended hand can lead to a handshake or other actions, and this suggests that in our everyday interactions the automatic and rapid decoding of social cues influences our intentional behavior, maximizing the efficiency of our responses. It is widely accepted that during action observation, the specific networks subserving that particular movement are already tuned for action (Fadiga et al., 2005). But the present results demonstrate that even observing congruent stimuli presented on a video display can have a measurable interference effect on simultaneously executed actions, depending on the context. The precise nature of this effect depends on the type of action presented in the video stimuli, with interference found for observation of a complementary request, and to a less degree for a non-interactive action. A possible explanation for our data comes from the hypothesis of a competition between different representations (Schubö et al., 2001; Dolk et al., 2014). According to these authors, the representations that underlie perceptual and motor activities, such as producing a movement while concurrently encoding an independent stimulus motion, must be “kept separate” so that the two activities can be carried out without interfering. In our study, we found a different degree of motor interference on the latency of Time to Peak Grip Velocity ranging from the non-interactive (2% delay) to the interactive (3% delay) conditions, despite the observed and executed movements were similar. Interestingly, the higher interference on grip aperture was connected to the planning of a complementary movement, thus suggesting a higher degree of competition between different representations.

### Response Competition and Inhibition Processes

In terms of grasping, an interference on the amplitude of Maximum Grip Aperture was specifically detected at 50% of movement execution. The direction of this effect (i.e., a decreased Grip Aperture) could be the byproduct of an automatic inhibition of representational features related to the complementary response (e.g., see Prinz and Hommel, 2002), in line with previous literature pointing to a bi-phasic pattern of interference of perception on ongoing action: initial assimilation followed by contrast (Dijkerman and Smit, 2007; Grosjean et al., 2009). Schubö et al. (2001) proposed that the representations that underlie distinct activities, such as producing a movement while concurrently encoding a perceptual event, must be “kept separate” so that the two activities can be carried out without interfering. The mechanism in question would involve a form of inhibition (Tipper et al., 1997) of the features shared by perception and action. Many models have thenceforth accounted for inhibition by referring to mechanisms associated with response competition (Swinnen, 2002; Duque et al., 2010, 2012; van den Berg et al., 2011; Verstynen and Ivry, 2011; Klein et al., 2012; Labruna et al., 2014). Since inhibition reflects the operation of a process involved in resolving response competition, here planning a whole-hand response had a repulsive effect on what was produced, decreasing the Maximum Grip Aperture and shifting the Trajectory path leftward (i.e., in the opposite direction with respect to the object requiring a whole-hand grasp).

### Maximum Grip Aperture

In this study we did not expect any change in Maximum Grip Aperture, since the same movement (i.e., a PG) was always repeated within the task and grip amplitude is known to covary linearly with object size (Jeannerod et al., 1995). The results were in line with our expectations. *Post hoc* analyses revealed that grip aperture remained constant throughout the experiment and well calibrated to the object size. Interestingly, the interactive request did not resulted in greater uncertainty in the performance of the participant’s grasping movement, since when subjects are uncertain during the grasp, they open their hand wider (Paulignan et al., 1991a,b). We may therefore assume that the preservation of maximal grip aperture across conditions is evidence that participants were confident in the movement to be executed.

### Motor Facilitation

Observing a congruent movement did not facilitate movement execution (Kilner et al., 2003; Bouquet et al., 2007; Stanley et al., 2007). This is probably due to the fact that observation of a congruent grasping action during execution of a similar action facilitates precision of the grasp component only if the two events are *highly synchronized* (Ménoret et al., 2013). Here, participants were asked to perform their movement at their own pace and no instruction was given concerning the speed of the movement. Moreover, the movement observed in the non-interactive condition had an additional level of complexity due to pouring the sugar in a cup as compared to the instructed movement of the participant. This could have also played a role in activating a partial competition between different representations.

### The Social Associative Memory Hypothesis

Overall, this study provides evidence that online interference occurs when an observed movement requires an incongruent grasping with respect to the prehension simultaneously observed and executed. This result, together with recent TMS studies on cortico-spinal excitability (Sartori et al., 2011b, 2012, 2013b,c) and previous kinematic data (Sartori et al., 2009), suggests that observing an interactive gesture automatically generates an internal *representation* of the required movement. Such an internal representation can cause interference in the execution of the grasping movement, when active at the same time.

An accumulating body of evidence seems to suggest the existence of a human *motor vocabulary* (Rizzolatti et al., 2001) in which congruent – and incongruent (Sartori et al., 2013a) – motor representations would be activated automatically during the observation of motor actions. According to Chinellato et al. (2013), a social associative memory would be in charge of matching certain actions to their natural social response, irrespective of who is actually performing the action. If action B (e.g., take) usually follows action A (e.g., give), the observation of a partner executing A elicits the pre-planning of B by the observer. On the other hand, if the subject executes A, she expects to see the partner performing B in response. The same concept has been put forward by Butterfill and Sinigaglia (2014): “Two outcomes, A and B, match in a particular context just if, in that context, either the occurrence of A would normally constitute or cause, at least partially, the occurrence of B or vice versa” (see also Catmur et al., 2009).

As in the case of previous literature on social Simon effect (Guagnano et al., 2010; Humphreys and Bedford, 2011; Dittrich et al., 2013), it remains to be clarified whether the social context is a necessary prerequisite or not for this interference effect. In this respect, a previous experiment with similar stimuli and an arrow indicating the target object instead of the social gesture suggested that the mere presence of an arrow pointing toward the object had the ability to determine MEP activation. However, such activity was reduced with respect to when the context was characterized by a request gesture toward the object (Sartori et al., 2011b). Those findings corroborate the idea that it is the social nature of the observed gesture, along with the presence of the object, to determine the observed effect.

## Conflict of Interest Statement

The authors declare that the research was conducted in the absence of any commercial or financial relationships that could be construed as a potential conflict of interest.
